# Editorial: Improving reproducibility in behavioral neuroscience

**DOI:** 10.3389/fnbeh.2023.1328525

**Published:** 2023-11-15

**Authors:** Cássio Morais Loss, Karolina Domingues, Nuno Sousa, Giordano Gubert Viola

**Affiliations:** ^1^Stiles-Nicholson Brain Institute, Charles E. Schmidt College of Medicine, Florida Atlantic University, Jupiter FL, United States; ^2^Molecular and Behavioral Neuroscience Laboratory, Departamento de Farmacologia, Universidade Federal de São Paulo, São Paulo, Brazil; ^3^Departmento de Anatomia, Instituto de Ciências Biomédicas, Universidade de São Paulo, São Paulo, Brazil; ^4^Life and Health Sciences Research Institute, School of Medicine, University of Minho, Braga, Portugal; ^5^Independent Researcher, Porto Alegre, Brazil

**Keywords:** replicability, reproducibility, behavior, data analysis, enriched environment, conditioned place preference, fear conditioning, automated systems

Reproducibility, sometimes referred to as replicability, is the process of obtaining consistent results across studies designed to answer the same scientific question, each of which obtained its own data. Replication, together with the design of experiments that permit the collection of disconfirmatory evidence, are cornerstones of the scientific method.

Importantly, when it comes to studying behavior and behavior-related disorders, the lack of reproducibility across multiple attempts/studies may imply ethical issues pertaining to harm/benefit or risk/benefit analyses, which are central to the use of animals for research. As the behavioral neuroscience field relies on experimentation on living organisms (including rodents, fish, invertebrates, and non-human primates), multiple failures to replicate an experimental result may suggest that a great number of lives are wasted for little benefit. In addition, studying behavior-related disorders commonly implies inducing morphological, emotional, and/or cognitive disabilities in the animals, followed by exposure to stressful environmental contexts. Thus, improving reproducibility in the field is mandatory, not only to improve the research output's quality but also to reduce the unnecessary use of laboratory animals.

It has been suggested that a potential way to combat the “reproducibility crisis” is to combine principles of animal welfare with experimental rigor (Loss et al., 2021). For this, the scientific community should revisit the scientific method literature to ensure that the experimental designs to be executed are in compliance with legislation, guidelines, methodological rigor, and ethical principles in animal research. This Research Topic focuses on discussing measures and procedures that the scientific community should adopt to improve the reliability and translational potential of pre-clinical research.

Behavioral measurements, suitable statistical methods, and data analysis, as well as the conceptualization and limitations associated with the protocols adopted for studying behavior, are among the subjects discussed by Yates. After reviewing 193 articles, the author elegantly highlights that not only discrepancies in the conditioned place preference (CPP) protocols may alter one's interpretation of results but also that contrasting conclusions can be drawn when CPP data are quantified in different ways, even in cases in which researchers use nearly identical methods. The author proposes a novel approach for analyzing CPP data (an adjusted CPP score) that, according to him, can be consistently applied across studies to increase replicability and to reduce some of the limitations associated with existing methods. He also discusses the importance of other measures researchers should take to increase transparency and replicability in CPP experiments (such as presentation of raw data and a detailed description of the procedures and apparatus used in the experiments).

Timotius et al. discuss the advantages, limitations, and challenges of using automated systems in behavioral experiments. The authors review 91 published pre-clinical studies using a commercially available automated gait analysis system. According to them, automated gait systems may provide sensitive locomotor and gait abnormalities measurements (classified as temporal, spatial, support, coordination, print, and others) that were not possible to assess by using non-automated methods. The authors discuss how monitoring a new set of time-based parameters may facilitate the evaluation of gait parameters with relevance to the study of traumatic brain injury, stroke, sciatic nerve injury, spinal cord injury, Parkinson's disease, and ataxia. In contrast, they highlight that the identification of the relevant variables (among the hundreds of variables available) and publication bias, reporting bias, and the lack of detailed description of the procedures are challenges/limitations that may prevent replication of the results obtained from automated systems.

In “*The systemic effects of the enriched environment on the conditioned fear reaction*”, Grigoryan brings up a conceptualization of the functional behavioral control system, of the conditioned fear reaction, and of the environmental enrichment (EE) and discusses how all of them are connected. The author points out that the influence of the EE on behavior is complex and multilateral, simultaneously involving all the key components of this system. He proposes that raising/subjecting animals to EE weakens fear responses (in Pavlovian-based fear-conditioned paradigms) by affecting the strength of the CS-US associations in a similar way as latent inhibition does and that these effects are beneficial for the organism. According to the author, even considering the heterogeneity of EE protocols [e.g., age of animal, type of enrichment, duration of stay in the EE; which, according to others, do not increase variability in outcome measures (Bayne and Wurbel, 2014; Bailoo et al., 2018)], the beneficial effect of it on the fear responses should still be observed.

The importance of combining a variety of behavioral paradigms when assessing anxiety-like behavior in experimental models of mild traumatic brain injury (mTBI) is discussed by Tseitlin et al. They used a weight drop concussive head injury device to model mTBI in male adult ICR mice. Seven days post-injury, they subjected the mice to one of the following behavioral paradigms: elevated plus maze, open field, marble burying, light–dark box, or light spot test. They found that the behavioral phenotype discrepancies between the animals subjected to the mTBI model and the control animals were dependent on the behavioral paradigm. According to them, the behavioral phenotypes could be influenced by the features of the protocol applied, such as the light intensity, the duration of the exposure to the aversive stimulus, the apparatus, and the properties of the stressors used.

In this Research Topic, we aimed to promote a discussion regarding theoretical concepts and practical actions underlining reproducibility/replicability issues. The articles presented here brought up fundamental aspects of experimental science, such as the conceptualization of both the subject under investigation and the behavioral paradigms being used. Their importance for experimental designs, including data collection and analysis, and the environmental conditions for raising animals or running behavioral experiments were highlighted. The authors emphasized a common concern regarding the frequent occurrence of reporting issues in the existing literature (e.g., lack of clarity or detailed description of methods), resulting in difficulties in summarizing and interpreting the results obtained by different studies. The editors hope the readers will enjoy this Research Topic and that it will be useful and insightful for their research.

## Author contributions

CML: Conceptualization, Writing—original draft, Writing—review & editing. KD: Conceptualization, Writing—original draft, Writing—review & editing. NS: Writing—review & editing. GGV: Conceptualization, Writing—original draft, Writing—review & editing.

## References

[B1] Bailoo J.D. Murphy E. Boada-Sana M. Varholick J.A. Hintze S. Baussiere C.. (2018). Effects of cage enrichment on behavior, welfare and outcome variability in female mice. Front. Behav. Neurosci. 12, 232. 10.3389/fnbeh.2018.00232 30416435 PMC6212514

[B2] Bayne K. Wurbel H. (2014). The impact of environmental enrichment on the outcome variability and scientific validity of laboratory animal studies. Rev. Sci. Tech. 33, 273–280. 10.20506/rst.33.1.2282 25000800

[B3] Loss C.M. Melleu F.F. Domingues K. Lino-de-Oliveira C. Viola G.G. (2021). Combining animal welfare with experimental rigor to improve reproducibility in behavioral neuroscience. Front. Behav. Neurosci. 15, 763428. 10.3389/fnbeh.2021.763428 34916915 PMC8671008

